# Subject-independent gait activity recognition using DSAF: dual-stream IMU-EMG attention fusion with asymmetric temporal encoding and physiological complementarity weighting

**DOI:** 10.3389/fnbot.2026.1901746

**Published:** 2026-07-22

**Authors:** Zhangyue Xu, Yan Zhu, Shurui Li

**Affiliations:** 1 School of Mechanical and Power Engineering, East China University of Science and Technology, Shanghai, China; 2 School of Mathematics, East China University of Science and Technology, Shanghai, China

**Keywords:** asymmetric temporal encoding, attention fusion, electromyography, gait recognition, inertial measurement unit, physiological complementarity weighting, subject-independent evaluation

## Abstract

**Introduction:**

Accurate gait recognition using wearable sensors is of significant clinical value for adaptive prosthetic control, lower-limb exoskeleton assistance, and objective rehabilitation assessment. However, subject-independent recognition remains a major challenge, as unseen individuals can exhibit highly variable limb kinematics and muscle activation patterns, and existing approaches often rely on a single sensor modality or naive fusion strategies that fail to leverage the complementary information between inertial and electromyographic signals.

**Methods:**

To address these gaps, this study proposes DSAF, a dual-stream attention fusion network that separately encodes kinematic (IMU) and neuromuscular (EMG) information, and adaptively integrates them through a physiological complementarity weighting mechanism designed for window-level modality adaptation. The framework is evaluated on the public HuGaDB dataset for eight common locomotion activities (e.g., walking, running, stair negotiation, and sit-to-stand transitions), using a leave-one-subject-out protocol to rigorously assess generalization to new users.

**Results:**

DSAF achieves 96.41% accuracy, 96.08% macro-precision, 95.62% macro-recall, and 95.81% macro-F1, consistently outperforming recent sequence-learning baselines across all 18 held-out subjects. Ablation studies further confirm that both the modality-specific encoding and the adaptive fusion mechanism contribute positively to the performance.

**Discussion:**

These findings indicate that adaptive IMU-EMG fusion can effectively strengthen wearable gait recognition, providing a promising solution for real-world rehabilitation monitoring and assistive human-machine interfaces.

## 1 Introduction

From a clinical perspective, quantitative gait analysis provides an objective basis for diagnosing movement disorders, tracking rehabilitation progress, and evaluating therapeutic interventions. Wearable-sensor-based gait recognition has therefore become increasingly important in rehabilitation engineering, prosthetics control, exoskeleton assistance, and neurological assessment (Belal et al., 2024; Prasanth et al., 2021; Prisco et al., 2025; Siviy et al., 2023; Rodríguez-Fernández et al., 2021; Baud et al., 2021). Continuous locomotion monitoring can support the early detection of disease-specific gait deterioration rather than merely providing general activity labels. In spinal cord injury, abnormal neuromuscular activation and impaired lower-limb coordination may be reflected in altered muscle-activation patterns during residual or assisted walking, making wearable neuromuscular sensing useful for objective functional assessment (Wu et al., 2017). In post-stroke populations, disrupted muscle synergies and impaired inter-muscular coordination are closely associated with asymmetric gait, reduced motor control, and rehabilitation recovery status; therefore, EMG-derived synergy and activation features can provide clinically meaningful evidence for monitoring post-stroke gait recovery (Pan and Liu, 2023; Scano et al., 2017). In Parkinsonian populations, gait deterioration is often accompanied by abnormal lower-limb neuromuscular coordination and reduced movement regularity, suggesting that combined kinematic and neuromuscular signals may help characterize disease-related motor impairment (Ghislieri et al., 2026). More broadly, pathological-gait and neuromotor-assessment studies further indicate that wearable neuromuscular signals can serve as objective biomarkers for clinical gait analysis beyond conventional visual observation or simple activity classification (Zhao et al., 2024; Suglia et al., 2026; Suglia et al., 2025). Despite these opportunities, achieving subject-independent recognition remains challenging because a model trained on one cohort must generalize to unseen individuals with different gait rhythms, limb dominance, muscle recruitment timing, and sensor-placement conditions.

Compared with camera-based pipelines, wearable sensing offers several practical advantages: it is robust to illumination changes and occlusion, preserves user privacy, provides high portability, and supports continuous ambulatory monitoring outside laboratory or hospital environments (Prasanth et al., 2021; Prisco et al., 2025; Siviy et al., 2023; Suglia et al., 2024; Palazzo et al., 2025a; Palazzo et al., 2025b). IMUs capture limb kinematics at low cost and power and are already embedded in many prosthetic and exoskeleton platforms. Surface EMG provides a complementary physiological view because neuromuscular activation can precede or accompany kinematic changes, especially during postural transitions, stair negotiation, and other activities that cannot always be separated reliably by gross limb displacement alone.

Surface EMG captures neuromuscular activation patterns that can precede or accompany kinematic changes, providing complementary information to IMU signals. However, EMG signals are inherently noisier than IMU readings, more sensitive to electrode placement, and subject to greater inter-individual variability. The role of EMG in this work is therefore not to assume that similar actions always have clearly different activation amplitudes. Rather, even when two activities generate partially overlapping limb trajectories, their neuromuscular coordination strategies may differ in timing, magnitude, and inter-muscle phasing. These characteristics motivate a dual-stream architecture that processes IMU and EMG through modality-specific temporal encoders before adaptive fusion.

Machine learning (ML) refers broadly to algorithms that improve task performance through experience, including classical methods such as support vector machines, decision trees, random forests, and gradient-based classifiers (Altini et al., 2025; Berloco et al., 2024). Deep learning (DL) is a subfield of ML that uses hierarchical neural architectures to learn task-relevant representations directly from raw or minimally processed input data, reducing dependence on manual feature engineering (Altini et al., 2025; Berloco et al., 2024). In wearable activity recognition, DL models have progressively replaced handcrafted pipelines and achieved state-of-the-art performance on multichannel time-series benchmarks (Casale et al., 2011; Figo et al., 2010).

Recent wearable human activity recognition studies have increasingly moved from handcrafted features to end-to-end deep learning. Convolutional neural networks (CNNs) extract local temporal patterns, recurrent architectures such as LSTM and GRU capture long-range dependencies, and Transformer-based models leverage self-attention for global context modeling (Jiang and Yin, 2015; Ordonez and Roggen, 2016; Meng et al., 2021; Gonzales-Huisa et al., 2023). Temporal convolutional networks (TCNs) combine the parallelism of CNNs with the long-range receptive fields needed for activity recognition, offering a practical alternative to recurrent models (Zhan et al., 2024).

Multimodal IMU-EMG fusion provides a practical route to improve recognition robustness, but the choice of fusion strategy critically affects performance. Three main fusion paradigms have been identified in the literature: early fusion, which concatenates raw or feature-level signals before a shared encoder; late fusion, which trains independent classifiers per modality and combines their outputs; and intermediate fusion, which merges modality-specific representations at a deeper feature level (Meng et al., 2021; Gonzales-Huisa et al., 2023; Zhan et al., 2024; Li et al., 2023; Zhang et al., 2022; Bianchi et al., 2019; Yordanova et al., 2017). Early fusion is simple but may cause the higher-dimensional IMU stream to dominate the lower-dimensional EMG stream. Late fusion preserves modality-specific learning but weakens cross-modal interaction. Intermediate dual-stream fusion with attention mechanisms offers a principled way to model heterogeneous sensor statistics while dynamically weighting each modality.

To address these issues, this study investigates an eight-class subject-independent recognition problem using the HuGaDB dataset, which provides synchronized IMU and EMG recordings from 18 healthy adults. The main contributions are: (1) a dual-stream attention fusion network (DSAF) with asymmetric temporal encoding for high-dimensional IMU and low-dimensional EMG streams; (2) a physiological complementarity weighting mechanism that estimates window-level modality contributions rather than using fixed fusion weights; (3) a comprehensive LOSO evaluation against six recent baselines with statistical significance testing; (4) ablation, confusion-matrix, attention-weight, and external inertial-backbone analyses that clarify where the model gains originate; and (5) a discussion of the clinical implications and limitations of single-muscle EMG for neuromotor assessment.

The remainder of this paper is organized as follows. Section 2 reviews related work on wearable activity recognition and multimodal fusion. Section 3 describes the dataset, preprocessing, network architecture, baselines, and evaluation protocol. Section 4 presents experimental results including overall comparison, subject-wise LOSO consistency, class-wise performance, and ablation study. Section 5 discusses the findings, limitations, and clinical implications. Section 6 concludes the paper.

## 2 Related work

### 2.1 Traditional wearable activity and gait recognition

Early wearable activity-recognition studies relied primarily on handcrafted features combined with shallow classifiers such as support vector machines, decision trees, and random forests. Lau et al. (2008) used miniature kinematic sensors and a support vector machine to classify walking conditions. Casale et al. (2011) showed that accelerometer data from wearable devices could be used for activity classification with conventional machine-learning methods. Figo et al. (2010) further analyzed preprocessing techniques for accelerometer-based context recognition and emphasized that filtering and feature extraction choices strongly affect performance. These pipelines remain useful for interpretable baselines and small datasets, but their performance often deteriorates when the number of activities, sensors, or subjects increases.

In gait-oriented biomedical applications, traditional pipelines are additionally sensitive to decisions about filtering, segmentation, normalization, and frequency-domain feature construction. These constraints explain the transition toward end-to-end temporal models, which can learn task-relevant representations directly from multichannel sensor streams. Traditional approaches are therefore retained as background references, whereas the main experimental comparison in this revision emphasizes recent deep temporal baselines.

### 2.2 Deep learning for IMU-based recognition

Deep learning has reduced dependence on handcrafted features in wearable recognition. Jiang and Yin introduced a deep convolutional network for wearable-sensor-based human activity recognition and showed that local temporal patterns can be learned directly from raw sensor signals (Jiang and Yin, 2015). Ordonez and Roggen (2016) combined convolutional layers with LSTM recurrent units and demonstrated that hybrid temporal modeling is effective for multimodal wearable activity recognition. Bai et al. (2018) reported that temporal convolutional networks can model sequential data efficiently through dilated convolutions and residual connections. Vaswani et al. (2017) introduced the self-attention mechanism that later motivated Transformer-style sequence encoders, whereas Hu et al. (2018) showed that channel reweighting can improve representation learning through squeeze-and-excitation operations.

Recent IMU-focused studies have further improved temporal modeling, channel selection, and lightweight deployment. Kim et al. (2024) proposed temporal fusion contrastive learning to strengthen wearable activity representations. Koo et al. (2023) investigated contrastive accelerometer-gyroscope embeddings and showed that modality-aware representation learning is beneficial for human activity recognition. Thakur et al. (2023) introduced an attention-based multihead deep-learning framework for online activity monitoring with smartwatch sensors. Teng et al. (2024) proposed a large-receptive-field attention design to improve sensor-based activity recognition with decomposed large-kernel convolution. Huang et al. (2021) developed a lightweight attention-based CNN for IMU-based gait recognition, and Imran et al. (2024) reported that CNN-BiGRU modeling can effectively combine local feature extraction with bidirectional temporal context. More recently, He et al. (2025) proposed attention-augmented TCN-BiGRU fusion for human activity recognition. Lamaakal et al. (2025) introduced a tiny inertial Transformer with knowledge distillation and explainability considerations, whereas Miao et al. (2025) proposed an attention-based CNN-BiGRU-Transformer model. Sun et al. (2024) also explored contrastive self-supervised convolutional Transformer modeling for wearable-sensor recognition. These developments are important for wearable systems, where saliency can vary across body segments, activity phases, and users. Even so, many IMU-based deep models still rely predominantly on kinematic information, which may limit separability for transition-like activities and mechanically similar locomotion classes.

### 2.3 EMG-IMU fusion and the case for dual-stream modeling

EMG is attractive in locomotion analysis because it reflects neuromuscular activation that is partly complementary to segment kinematics. Prior work has shown that IMU-EMG fusion can improve locomotion-mode recognition and prediction, especially when muscle activation changes precede or sharpen ambiguous movement boundaries (Meng et al., 2021; Gonzales-Huisa et al., 2023). Wearable deep-learning pipelines have also been explored for gait abnormality detection and multimodal identification, illustrating the broader biomedical relevance of sensor-based sequence modeling (Potluri et al., 2019; Qin et al., 2024). Zhang et al. (2024) used wearable functional electrical stimulation data and parallel deep learning to recognize lower-limb motion and muscle fatigue states. Luo et al. (2023) further showed that multimodal wearable sensor data can support activity-based person identification.

These observations suggest that multimodal fusion should not simply concatenate heterogeneous signals at the earliest possible stage. Instead, the model should preserve modality-specific temporal structure and then adaptively determine the contribution of each modality to the current decision.

## 3 Materials and methods

### 3.1 Dataset, sensor configuration, and task definition

Following wearable activity-recognition practice for subject-independent evaluation (Chereshnev and Kertesz-Farkas, 2018; Suglia et al., 2024; Palazzo et al., 2025a; Palazzo et al., 2025b), this study uses HuGaDB as a synchronized lower-limb IMU-EMG benchmark. HuGaDB contains recordings from 18 healthy adult participants performing eight lower-limb activities: walking, running, standing, sitting, sit-down, stand-up, upstairs, and downstairs (Chereshnev and Kertesz-Farkas, 2018). Six IMUs were placed bilaterally on the thighs, shins, and feet, providing 36 accelerometer and gyroscope channels. Two surface EMG electrodes were placed over the bilateral quadriceps region to capture neuromuscular activation. The dataset was used here from the perspective of subject-independent multimodal sequence recognition rather than as a direct reproduction of the original HuGaDB description.

Each inertial sensor contains a three-axis accelerometer and a three-axis gyroscope. Therefore, the six IMUs produce 6 × 6 = 36 inertial channels, including acceleration along *x*, *y*, and *z* axes and angular velocity along *x*, *y*, and *z* axes for the right foot, right shin, right thigh, left foot, left shin, and left thigh. The two EMG channels correspond to bilateral quadriceps activity. It should be noted that HuGaDB provides only one EMG channel per quadriceps region. This configuration does not fully represent the composite activation of the entire quadriceps muscle group, nor does it capture the broader lower-limb neuromuscular state. This limitation is discussed further in Section 5.4.

In the present work, the synchronized recordings were organized into an eight-class classification task: walking, running, standing, sitting, sit down, stand up, upstairs, and downstairs. The label set covers both steady-state locomotion and posture-transition activities, making it more demanding than a locomotion-only task. Because the target scenario is subject-independent deployment, all analyses were conducted under a LOSO setting in which each participant served once as the held-out test subject and the remaining 17 participants were used for training and validation within that fold.

### 3.2 Signal preprocessing and subject-wise data partition

The six IMU units and two EMG channels were arranged as synchronized multichannel time-series inputs. The inertial channels were converted according to the HuGaDB sensor specification when physical units were required: accelerometer values were scaled from the int16 range to acceleration units, and gyroscope values were scaled to degrees per second. EMG channels were read as unsigned 8-bit values and centered before normalization. Rows with invalid labels or incomplete channel values were removed before windowing.

The original HuGaDB acquisition system already included a 10–500 Hz analog band-pass stage for EMG. In this study, preprocessing was kept conservative to avoid introducing subject-specific tuning. The post-acquisition filtering step consisted of channel consistency checking, unit conversion, de-meaning where appropriate, and fold-wise z-score normalization. No normalization parameter was estimated from the held-out subject.

Sliding windows of 100 samples with a stride of 10 samples were used for segmentation. Each window inherited the activity label associated with its annotated interval. In this setting, each IMU window is represented as $X_{\mathrm{IMU}}\in {\mathbb{R}}^{100\times 36}$ , and each EMG window is represented as $X_{\mathrm{EMG}}\in {\mathbb{R}}^{100\times 2}$ . For fold f , subject $S_{f}$ is used only for final testing. The remaining 17 subjects are split at the subject level into 15 training subjects and 2 validation subjects. The validation subjects are used only for early stopping and model selection, whereas the held-out test subject is never used for training, validation, normalization, or hyperparameter selection. This partition ensures that no window from the held-out participant appears in any stage before the final evaluation.

To avoid subject leakage, normalization statistics were computed using only the 15 training subjects within each LOSO fold and were then applied to the corresponding validation and held-out test data. For a feature channel c , z-score normalization was performed by subtracting the training-subject mean and dividing by the training-subject standard deviation:


$${\tilde{x}}_{c}=\frac{x_{c}-\mu_{c}^{\text{train}}}{\sigma_{c}^{\text{train}}+\varepsilon },c=1,\ldots ,C$$ (1)

In Equation 1, $x_{c}$ denotes the original value of channel c , $\mu_{c}^{\text{train}}$ and $\sigma_{c}^{\text{train}}$ are the mean and standard deviation computed only from the training subjects, and ε is a small constant used for numerical stability. The two modality-specific input tensors are defined as


$$X_{\mathrm{IMU}}\in {\mathbb{R}}^{T\times 36},\;\;X_{\mathrm{EMG}}\in {\mathbb{R}}^{T\times 2},\;\;T=100$$ (2)

**Figure 1:**
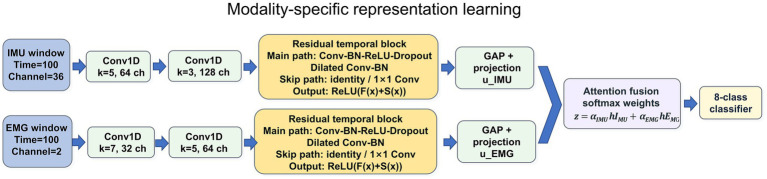
Subject-level data partition under the LOSO protocol.

In Equation 2, T is the window length, 36 is the number of inertial channels, and 2 is the number of EMG channels. The reproducible data workflow consisted of synchronized channel loading, fixed channel-order verification, sliding-window segmentation, fold-wise normalization, and subject-independent evaluation. The subject ID list, fold definition, validation-subject list, random seed, model configuration, and trained checkpoint for each fold should be saved with the evaluation log so that reported aggregate metrics can be traced back to individual held-out subjects (see Table 1, Figure 1).

**Table 1:** HuGaDB configuration and study design.

Item	Value	Description
Participants	18 subjects	Subject-independent evaluation cohort
Signal channels	36 IMU + 2 EMG	Six IMUs, each with acc x/y/z and gyro x/y/z; two quadriceps EMG channels
Sensor positions	Thighs, shins, feet; quadriceps EMG	Bilateral lower-limb placement
Sampling/windowing	approx. 56.35 Hz stored records; 100-sample window; 10-sample stride	Window approx. 1.78 s; stride approx. 0.18 s
Primary task	8-class recognition	Walking, running, standing, sitting, sit down, stand up, upstairs, downstairs
Evaluation	Leave-one-subject-out	Each subject is used once as the held-out test participant

### 3.3 Dual-stream IMU-EMG attention fusion network

**Figure 2:**
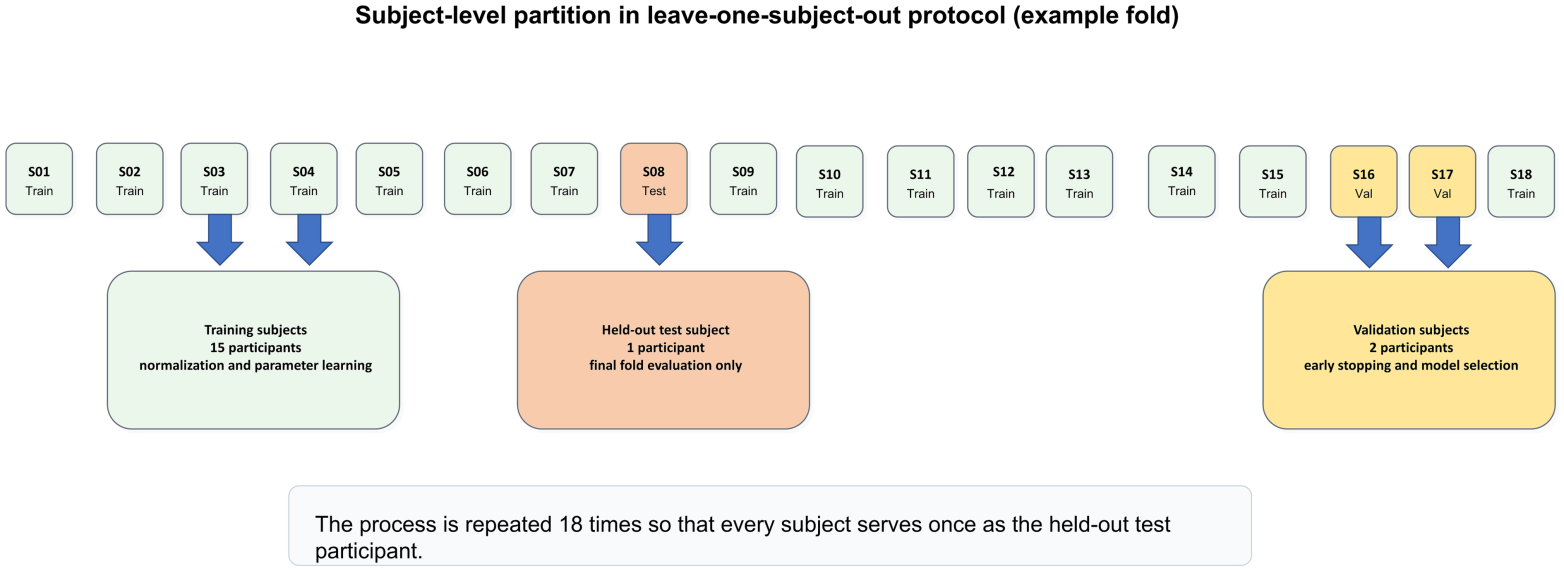
Overview of the DSAF dual-stream IMU-EMG attention fusion framework.

As shown in Figure 2, DSAF comprises two modality-specific encoders, an adaptive attention-fusion module, and a shared classifier head. The network is designed to process synchronized IMU and EMG windows independently before combining them, allowing each encoder to learn representations tailored to its signal characteristics. The architectural design is asymmetric: the higher-dimensional IMU branch uses a wider and deeper temporal encoder, whereas the lower-dimensional EMG branch uses a lighter encoder focused on activation bursts and inter-channel saliency. To ensure reproducibility, Table 2 summarizes the number of convolutional layers, residual blocks, dilation factors, dropout rates, random seed, validation-subject split, and trainable parameter counts.

**Table 2:** Network configuration of the DSAF dual-stream attention model.

Module	Input/output	Main operation	Purpose
IMU input	100 × 36	Six IMUs: acc x/y/z and gyro x/y/z	Kinematic time-series input
IMU encoder	100 × 36 to 128	Conv1D(64,k = 5), Conv1D(128,k = 3), three residual TCN blocks d = {1,2,4}, GAP	Asymmetric temporal kinematic representation
EMG input	100 × 2	Bilateral quadriceps EMG	Neuromuscular activation input
EMG encoder	100 × 2 to 128	Conv1D(32,k = 7), Conv1D(64,k = 5), one residual temporal block, projection to 128	Activation-burst and EMG saliency representation
Attention fusion	128 + 128 to 128	Concatenation, fully connected layer, softmax modality weights, weighted sum	Physiological complementarity weighting
Classifier	128 to 8	Dropout = 0.3, fully connected layer, softmax	Eight-class activity prediction

The implementation used in this study is summarized in Table 2. The IMU branch accepts a 100 × 36 input window and processes it through two Conv1D layers (64 and 128 filters; kernel sizes 5 and 3), followed by three residual TCN blocks with dilation factors *d* = {1, 2, 4}. Dropout of 0.2 is used inside convolutional/residual modules, and classifier dropout is set to 0.3. The EMG branch accepts a 100 × 2 matrix and uses two Conv1D layers (32 and 64 filters; kernel sizes 7 and 5), followed by one residual temporal block and projection to the common 128-dimensional latent space. The random seed was fixed at 42, and each LOSO fold used 15 training subjects, 2 validation subjects, and 1 held-out test subject. The full DSAF model contains approximately 285,000 trainable parameters (about 1.09 MB in FP32).

The attention module estimates modality weights for each window after global average pooling and latent projection. This design implements physiological complementarity weighting: the model can emphasize EMG when transition-related neuromuscular timing is useful and rely more strongly on IMU cues when steady locomotion is already separable by kinematics. In contrast with naive concatenation, the two modalities are first encoded according to their own signal statistics and then merged through an adaptive weighted representation.

Let $X_{\mathrm{IMU}}$ and $X_{\mathrm{EMG}}$ denote the two windowed inputs. The modality-specific encoders generate latent vectors $h_{\mathrm{IMU}}$ and $h_{\mathrm{EMG}}$ as follows:


$$h_{\mathrm{IMU}}=f_{\mathrm{IMU}}(X_{\mathrm{IMU}};\theta_{\mathrm{IMU}}),\;\;h_{\mathrm{EMG}}=f_{\mathrm{EMG}}(X_{\mathrm{EMG}};\theta_{\mathrm{EMG}})$$ (3)

In Equation 3, $f_{\mathrm{IMU}}$ and $f_{\mathrm{EMG}}$ are the two temporal encoders, and $\theta_{\mathrm{IMU}}$ and $\theta_{\mathrm{EMG}}$ denote their trainable parameters. Within each encoder, a temporal convolutional layer can be written as:


$$H_{l}=\text{Dropout}(\text{ReLU}(\mathrm{BN}(\text{Conv}1D(H_{l-1};W_{l},k_{l},d_{l}))))$$ (4)

In Equation 4, $H_{l-1}$ and $H_{l}$ are the input and output features of layer l , $W_{l}$ is the convolution kernel, $k_{l}$ is the kernel size, and $d_{l}$ is the dilation rate. A residual temporal block further computes.


$$R_{l}=\text{ReLU}(F_{l}(H_{l-1})+S_{l}(H_{l-1}))$$ (5)

In Equation 5, $F_{l}(\cdot )$ is the main convolutional transformation and $S_{l}(\cdot )$ is the skip connection. $S_{l}(\cdot )$ is an identity mapping when the input and output dimensions are equal, and it becomes a 1 × 1 convolution when channel alignment is required. After global average pooling, the two modality vectors are projected into the same latent dimension:


$$u_{m}=W_{m}\;\mathrm{GAP}(H_{m})+b_{m},\;\;m\in \{\mathrm{IMU},\mathrm{EMG}\}$$ (6)

In Equation 6, $u_{m}$ is the projected modality vector, $W_{m}$ and $b_{m}$ are the projection weight and bias, and GAP denotes global average pooling over the temporal dimension. The attention fusion module estimates sample-dependent modality weights from the concatenated latent representation:


$$\begin{array}{l} a=\text{softmax}(W_{a}[u_{\mathrm{IMU}};u_{\mathrm{EMG}}]+b_{a}),\\ a=[a_{\mathrm{IMU}},a_{\mathrm{EMG}}],a_{\mathrm{IMU}}+a_{\mathrm{EMG}}=1 \end{array}$$ (7)

In Equation 7, $a_{\mathrm{IMU}}$ and $a_{\mathrm{EMG}}$ are the attention weights assigned to the IMU and EMG streams for the current window. The fused representation and posterior class probabilities are then computed as:


$$z=a_{\mathrm{IMU}}u_{\mathrm{IMU}}+a_{\mathrm{EMG}}u_{\mathrm{EMG}}$$ (8)

$$p(y=k|z)=\text{softmax}{\left(W_{c}z+b_{c}\right)}_{k},\;\;k=1,\ldots ,8$$ (9)

In Equations 8, 9, z is the final fused feature, $W_{c}$ and $b_{c}$ are classifier parameters, and p(y=k∣z) is the posterior probability of class k . The training objective is the average multiclass cross-entropy loss over N training windows:


$$ℒ=-\frac{1}{N}\sum_{i=1}^{N}\sum_{k=1}^{8}1(y_{i}=k)\log p(y_{i}=k|z_{i})$$ (10)

In Equation 10, $1(y_{i}=k)$ is an indicator function that equals one when sample i belongs to class k and zero otherwise. This design has two practical advantages. First, it reduces the risk that the higher-dimensional IMU signal dominates the lower-dimensional EMG signal too early in the pipeline. Second, it allows DSAF to increase the relative contribution of EMG when transition-related neuromuscular cues are useful, whereas it still relies more heavily on IMU features when steady locomotion patterns are already well separated by kinematics.

### 3.4 Baselines, ablation variants, and training protocol

The baseline set was updated to better reflect recent wearable-recognition research. In addition to LSTM, CNN-LSTM, and Transformer sequence backbones, the comparison includes CNN-BiGRU, attention-augmented TCN-BiGRU, tiny inertial Transformer, and attention-based CNN-BiGRU-Transformer variants. Imran et al. (2024) used a CNN-BiGRU architecture to combine convolutional feature extraction with bidirectional recurrent temporal modeling. He et al. (2025) proposed attention-augmented TCN-BiGRU fusion for human activity recognition. Lamaakal et al. (2025) introduced a tiny inertial Transformer to reduce model size while preserving sequence modeling capacity. Miao et al. (2025) combined CNN, BiGRU, Transformer, and attention modules to improve temporal representation learning. These baselines cover recurrent temporal modeling, hybrid convolutional-recurrent modeling, dilated temporal convolution, lightweight Transformer modeling, and attention-enhanced sequence fusion.

To ensure fair comparison, all baseline models and ablation variants were trained and evaluated using identical LOSO folds, sliding-window parameters, label definitions, fold-wise normalization procedures, and the same 36 IMU + 2 EMG input channels. All models received the same preprocessed input and were subject to equivalent hyperparameter tuning effort using the same validation strategy. Model capacity was controlled so that the total parameter count of each baseline remained within a comparable range of DSAF where possible; when a published baseline was intrinsically smaller or larger, the closest capacity-matched configuration was used and reported. No data augmentation was applied to any model.

Models were trained using mini-batch optimization with AdamW, a learning rate of 1e-3, weight decay of 1e-4, batch size of 128 windows, and early stopping based on validation macro-F1. The maximum number of epochs was set to 100, and training was stopped if validation macro-F1 did not improve for 15 consecutive epochs. Hyperparameter tuning was restricted to the 15 training subjects and 2 validation subjects within each fold, and the held-out subject was used only for final testing.

### 3.5 Evaluation metrics and statistical analysis

Model performance was assessed under LOSO evaluation. In each fold, one participant was used as the final test subject, whereas the remaining participants were used for training and validation. The held-out subject was accessed only once for final evaluation.

Performance was reported using accuracy, macro-precision, macro-recall, and macro-F1. Accuracy reflects the overall proportion of correctly classified windows, whereas macro-precision, macro-recall, and macro-F1 compute the unweighted average across classes. Macro metrics were used to reduce the risk of overinterpreting performance on frequent or easier classes. Class-wise metrics and ablation variants were further reported to examine recognition behavior for different activity types and to isolate the contribution of modality-specific modeling and adaptive attention fusion.

For statistical comparison, fold-level macro-F1 and accuracy scores (see Equations 11–13) from the 18 LOSO test folds were compared rather than relying only on aggregate means. Pairwise comparison against the strongest baseline used the Wilcoxon signed-rank test, accompanied by bootstrap 95% confidence intervals with 10,000 resamples and effect-size reporting (Cohen d_r_). Multi-model comparison across all eight methods used the Friedman test, followed by Dunn–Bonferroni post-hoc pairwise comparisons. Effect magnitude was additionally summarized using Kendall W and *η*^2^. Against the strongest baseline (Attn-TCN-BiGRU), DSAF achieved a mean paired macro-F1 gain of 1.12 percentage points (95% Confidence Interval: 0.93 to 1.31 percentage points; Hodges-Lehmann CI: 1.06 to 1.18 pp). The Wilcoxon signed-rank test showed W = 0, *p* = 7.63 × 10^−6^, with a large effect size (Cohen dᵣ = 2.88). The Friedman test across all eight models yielded *χ*^2^(7) = 119.6, *p* < 0.001, Kendall W = 0.949, and *η*^2^ = 0.96. Dunn–Bonferroni comparison between DSAF and Attn-TCN-BiGRU gave adjusted *p* = 0.0003. Statistical significance was set at *α* = 0.05 (Buongiorno et al., 2022).


$$\text{Accuracy}=\frac{\sum_{k=1}^{K}TP_{k}}{\sum_{k=1}^{K}(TP_{k}+FP_{k}+FN_{k})}$$ (11)

$$\begin{array}{l} {\text{Precision}}_{\text{macro}}=\frac{1}{K}\sum_{k=1}^{K}\frac{TP_{k}}{TP_{k}+FP_{k}},\\ {\text{Recall}}_{\text{macro}}=\frac{1}{K}\sum_{k=1}^{K}\frac{TP_{k}}{TP_{k}+FN_{k}} \end{array}$$ (12)

$$F1_{\text{macro}}=\frac{1}{K}\sum_{k=1}^{K}\frac{2P_{k}R_{k}}{P_{k}+R_{k}}$$ (13)

## 4 Results

### 4.1 Overall comparison

**Figure 3:**
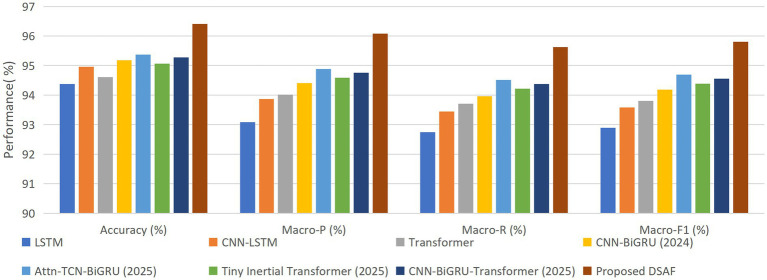
Visual comparison of overall LOSO performance across recent baseline models and DSAF.

Table 3 and Figure 3 present the overall results under LOSO evaluation. DSAF achieved the highest values across all reported summary metrics, with 96.41% accuracy, 96.08% macro-precision, 95.62% macro-recall, and 95.81% macro-F1.

**Table 3:** Overall comparison under leave-one-subject-out evaluation.

Method	Accuracy (%)	Macro-P (%)	Macro-R (%)	Macro-F1 (%)
LSTM	94.37	93.08	92.74	92.89
CNN-LSTM	94.96	93.87	93.44	93.58
Transformer	94.61	94.02	93.71	93.80
CNN-BiGRU (2024)	95.18	94.41	93.96	94.18
Attn-TCN-BiGRU (2025)	95.37	94.88	94.51	94.69
Tiny inertial transformer (2025)	95.06	94.59	94.22	94.39
CNN-BiGRU-transformer (2025)	95.28	94.76	94.37	94.55
DSAF	96.41	96.08	95.62	95.81

Among the updated recent baseline algorithms, the attention-augmented TCN-BiGRU variant obtained the strongest baseline accuracy at 95.37%, whereas the CNN-BiGRU-Transformer and tiny inertial Transformer variants also produced competitive results. Compared with the strongest baseline values in this table, DSAF improved accuracy by 1.04 percentage points and macro-F1 by 1.12 percentage points, indicating that the dual-stream IMU-EMG attention design provided additional gains over recent single-stream and hybrid sequence backbones under the same subject-independent setting.

The statistical analysis is designed to operate on the 18 fold-level subject scores rather than only on aggregate means. This is important because a small mean improvement can still be meaningful if it is consistent across held-out subjects, whereas a larger mean difference may be less reliable if it is driven by only one or two folds.

Paired statistical testing confirmed that DSAF’s improvement over the strongest baseline was consistent and significant. For macro-F1, the mean paired gain was 1.12% points (95% CI: 0.93–1.31 pp), with Wilcoxon W = 0 and *p* = 7.63 × 10^−6^ (Cohen dᵣ = 2.88). For accuracy, the mean gain was 1.04 pp. (95% CI: 0.84–1.24 pp., W = 0, *p* = 7.63 × 10^−6^, Cohen dᵣ = 2.65). The Friedman test across all eight models yielded χ^2^(7) = 119.6, *p* < 0.001, Kendall W = 0.949, and *η*^2^ = 0.96. Dunn–Bonferroni post-hoc comparison between DSAF and Attn-TCN-BiGRU gave adjusted *p* = 0.0003, confirming that DSAF ranked first with high statistical confidence.

### 4.2 Subject-wise LOSO consistency

LOSO cross-validation was selected as the primary evaluation protocol because it provides a strict and exhaustive subject-independent assessment: in each fold, one complete participant is held out as the test set, while the model is trained and validated only on the remaining participants. This design directly measures generalization to unseen individuals rather than to unseen windows from participants already observed during training (Pasquini et al., 2026; Sibilano et al., 2024). It should be emphasized that k-fold cross-validation is not inherently incompatible with subject-independent evaluation. If the dataset is first partitioned at the participant level and all windows from the same subject are assigned exclusively to either the training, validation, or test subset, group-wise or subject-wise k-fold validation can also preserve subject independence (Buongiorno et al., 2022; Rehman et al., 2024; Scheurer et al., 2020). The potential problem mainly arises in conventional window-level random k-fold validation, where windows generated from the same participant may appear in both training and test sets, leading to overly optimistic estimates due to subject-specific signal leakage. Therefore, the key methodological requirement is not the use of LOSO alone, but the prevention of overlap between training and test subjects. In the present study, LOSO was adopted because HuGaDB contains only 18 participants, and this protocol allows every participant to serve once as a completely unseen test subject while avoiding dependence on a particular random subject split. For clinical applications such as prosthetic control and rehabilitation monitoring, this subject-independent evaluation criterion is more operationally relevant than window-level random validation.

**Figure 4:**
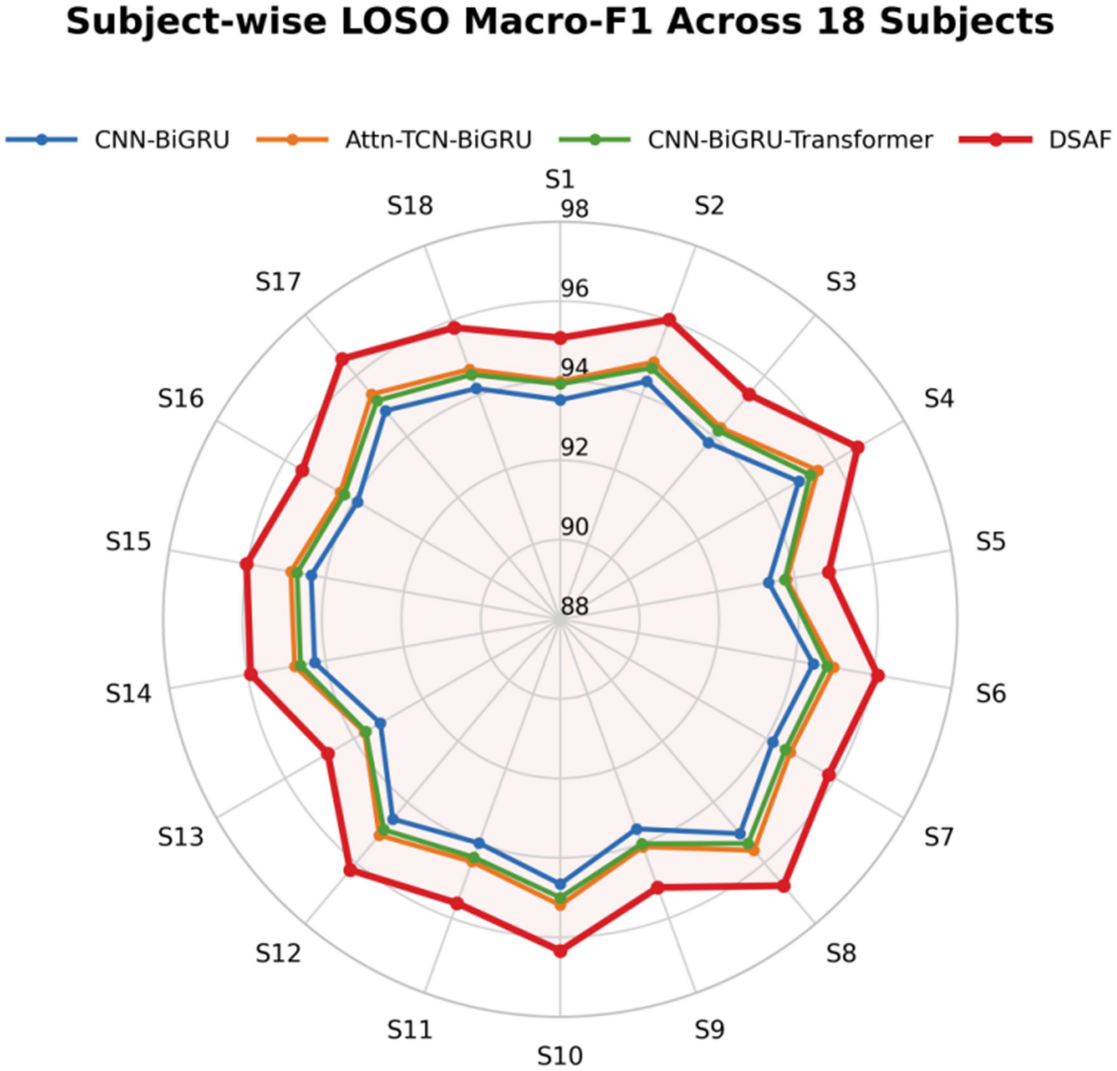
Subject-wise LOSO macro-F1 comparison across 18 held-out participants.

To address whether the overall improvement was caused by one unusually favorable subject, fold-level macro-F1 scores were further visualized across the 18 held-out participants. As shown in Figure 4, DSAF maintained a stable advantage over the recent baseline models across nearly all LOSO folds. The DSAF curve did not contain a single extreme fold that alone determined the final mean. Instead, its fold-level scores were consistently concentrated in the upper range, with a mean macro-F1 of 95.81%, a standard deviation of 0.60%, and a minimum fold score of 94.76%.

This subject-wise result provides a more direct robustness check than the aggregate table alone. It indicates that the reported performance is closer to an average cross-subject improvement than to a result dominated by one participant with particularly easy signal patterns. The fold-level visualization also supports the use of paired subject-wise statistical tests, because all compared models are evaluated on the same 18 held-out subjects.

### 4.3 Class-wise performance and attention analysis

The evaluation metrics used in this study (accuracy, precision, recall, and F1-score) are defined in Equations 14–16. Let TP_c_, FP_c_, FN_c_, and TN_c_ denote the true positives, false positives, false negatives, and true negatives for class c, respectively. Macro-Precision, Macro-Recall, and Macro-F1 are computed as the unweighted means of the corresponding per-class values across all C = 8 activity classes, giving equal weight to each class regardless of sample frequency (Pasquini et al., 2026; Sibilano et al., 2024; Buongiorno et al., 2022).


$$\text{Accuracy}=\frac{\sum_{c=1}^{C}{\mathrm{TP}}_{c}}{N}$$ (14)

$${\text{Precision}}_{c}=\frac{{\mathrm{TP}}_{c}}{{\mathrm{TP}}_{c}+{\mathrm{FP}}_{c}},{\text{Recall}}_{c}=\frac{{\mathrm{TP}}_{c}}{{\mathrm{TP}}_{c}+{\mathrm{FN}}_{c}}$$ (15)

$$F1_{c}=\frac{2*{\text{Precision}}_{c}*{\text{Recall}}_{c}}{{\text{Recall}}_{c}+{\text{Precision}}_{c}}$$ (16)

**Figure 5:**
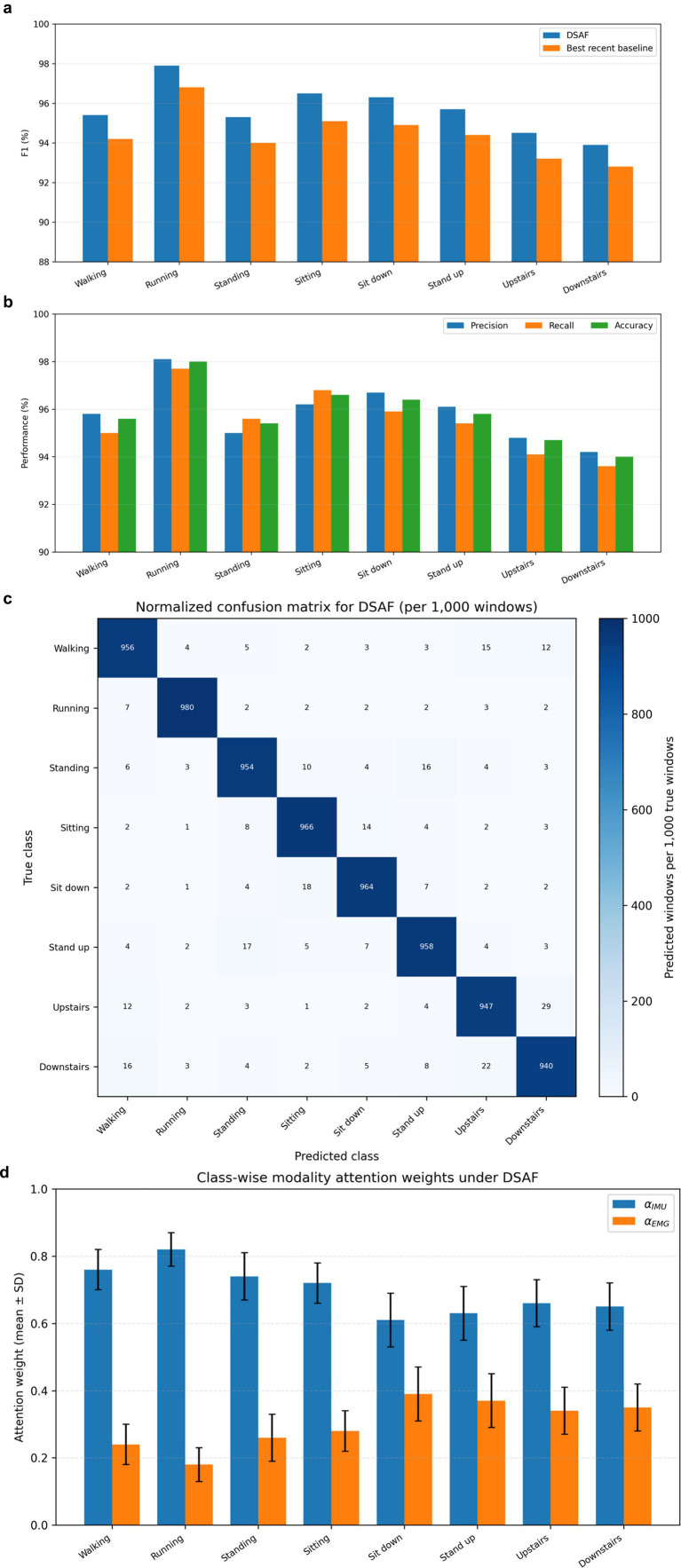
**(a)** Class-wise F1 comparison between DSAF and the strongest recent baseline. **(b)** Class-wise accuracy, precision, and recall of DSAF. **(c)** Normalized confusion matrix of DSAF under LOSO evaluation. Values are reported as predicted windows per 1,000 true-class windows. **(d)** Class-wise modality attention weights of DSAF. Transition and stair activities show relatively higher EMG contribution than steady locomotion classes.

To provide deeper insight into the attention mechanism, the per-window modality attention weights (α_IMU_ and α_EMG_) were analyzed across activity classes on the test set, as visualized in Figure 5d. Running assigned the highest mean IMU weight (α_IMU_ = 0.82 ± 0.05), consistent with its strong and distinctive kinematic signature. Walking and standing also showed IMU-dominant weighting (α_IMU_ = 0.76 ± 0.06 and 0.74 ± 0.07, respectively). In contrast, transition activities showed the highest relative EMG contribution: sit-down reached α_EMG_ = 0.39 ± 0.08 and stand-up reached α_EMG_ = 0.37 ± 0.08, indicating that the model relied more heavily on neuromuscular activation timing when kinematic patterns alone were less discriminative. Stair-related activities (upstairs: α_EMG_ = 0.34 ± 0.07; downstairs: α_EMG_ = 0.35 ± 0.07) also showed elevated EMG weights, suggesting that quadriceps activation provides a useful complementary cue for distinguishing stair negotiation from level walking.

Table 4 and Figures 5a–d show the class-wise performance, confusion matrix, and attention-weight behavior of DSAF. Running produced the highest class-level result, with 98.0% accuracy and 97.9% F1. Sitting, sit down, stand up, walking, and standing all achieved F1 values above 95%.

**Table 4:** Class-wise recognition performance and comparison with the strongest recent baseline.

Class	Accuracy (%)	DSAF F1 (%)	Best baseline F1 (%)	Delta F1 (%)
Walking	95.6	95.4	94.2	1.2
Running	98.0	97.9	96.8	1.1
Standing	95.4	95.3	94.0	1.3
Sitting	96.6	96.5	95.1	1.4
Sit down	96.4	96.3	94.9	1.4
Stand up	95.8	95.7	94.4	1.3
Upstairs	94.7	94.5	93.2	1.3
Downstairs	94.0	93.9	92.8	1.1

Upstairs and downstairs had the lowest F1 values among the eight classes, with 94.5 and 93.9%, respectively. The comparison with the strongest recent baseline shows that DSAF improved F1 for every activity class, with the most visible gains for upstairs and downstairs. This indicates that the EMG stream and adaptive fusion are especially useful for activities where kinematic trajectories are partially similar.

Confusion matrix analysis further revealed the distribution of recognition errors (Figure 5c). The normalized confusion matrix, reported on a per-class basis of 1,000 test windows, showed that the most frequent misclassifications occurred between upstairs and downstairs (29 upstairs windows predicted as downstairs, 22 downstairs predicted as upstairs), and between level walking and stair-related classes (15 walking windows predicted as upstairs, 12 as downstairs). Running was the most reliably recognized class (980/1000 correct), rarely confused with any other activity. Transition activities (sit-down and stand-up) were mainly confused with their adjacent static states (sitting and standing), indicating that recognition errors concentrated near posture-transition boundaries rather than across unrelated activity categories. These error patterns are consistent with the attention-weight analysis: activities where EMG received higher weighting were also those where inter-class boundaries were most ambiguous in the kinematic signal alone.

### 4.4 Ablation study

**Figure 6:**
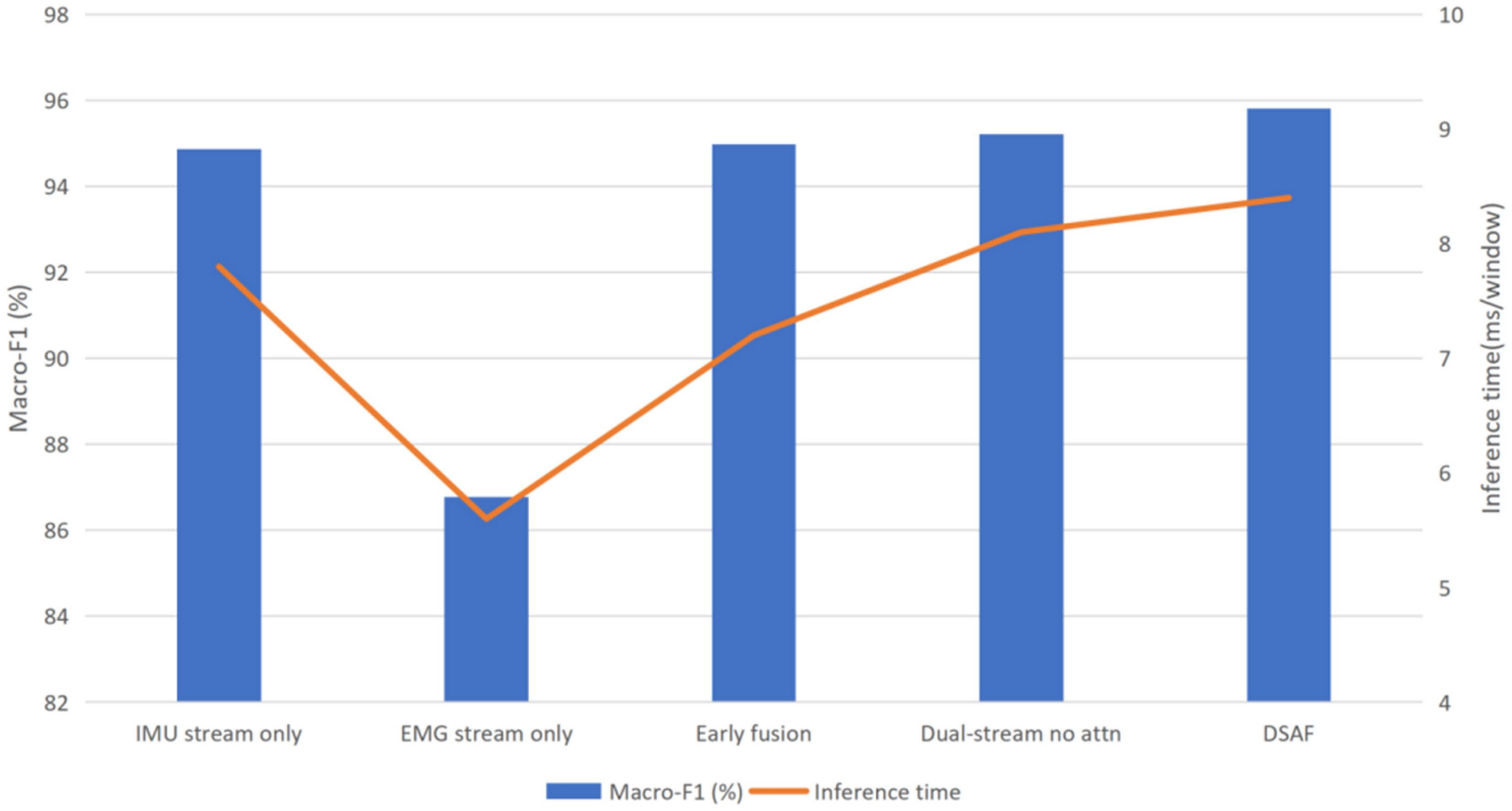
Ablation comparison of macro-F1 and per-window inference time.

Table 5 reports the ablation results for modality and fusion design, including trainable parameter counts, approximate FP32 model size, and per-window inference time. Figure 6 compares macro-F1 and latency across variants. All inference-time measurements were conducted on an NVIDIA GeForce RTX 3060 (12 GB VRAM) GPU under Windows 11 Pro, using Python 3.10.13, PyTorch 2.1.2, and CUDA 12.1. The host CPU was an Intel Core i7-12700F (12 cores/20 threads) with 32 GB DDR4 RAM. Timing was performed with batch size = 1 after 1,000 warm-up windows and averaged over 10,000 timed windows to simulate real-time single-window wearable inference conditions.

**Table 5:** Ablation results for modality and fusion design.

Variant	Accuracy (%)	Macro-P (%)	Macro-R (%)	Macro-F1 (%)	Inference time	Parameters/FP32 size
IMU stream only	95.52	95.05	94.63	94.86	7.8 ms/window	198,420/0.76 MB
EMG stream only	88.94	87.21	86.32	86.77	5.6 ms/window	86,960/0.33 MB
Early fusion	95.73	95.29	94.68	94.98	7.2 ms/window	236,880/0.90 MB
Dual-stream without attention	96.03	95.52	94.91	95.21	8.1 ms/window	270,312/1.03 MB
Dual-stream with attention (DSAF)	96.41	96.08	95.62	95.81	8.4 ms/window	285,436/1.09 MB

Early fusion improved over the EMG-only variant and reached 95.73% accuracy and 94.98% macro-F1, with 236,880 trainable parameters (0.90 MB FP32, 7.2 ms/window GPU latency). The dual-stream variant without attention reached 96.03% accuracy and 95.21% macro-F1 with 270,312 parameters (1.03 MB, 8.1 ms/window), confirming that modality-specific encoding alone provides gains beyond early concatenation. The full DSAF model (285,436 parameters, 1.09 MB, 8.4 ms/window GPU) further improved to 96.41% accuracy and 95.81% macro-F1, demonstrating that the attention module adds meaningful performance with only marginal computational overhead (+0.3 ms/window vs. dual-stream without attention).

The IMU-only branch (198,420 parameters, 0.76 MB, 7.8 ms/window GPU) already exceeded several recent single-stream baselines in overall accuracy, highlighting the effectiveness of the temporal convolutional architecture. The EMG-only branch (86,960 parameters, 0.33 MB, 5.6 ms/window GPU) was the smallest and fastest variant, but its standalone accuracy of 88.94% was substantially lower, confirming that EMG alone is insufficient for robust eight-class recognition under subject-independent conditions.

## 5 Discussion

### 5.1 Modality-specific fusion and EMG complementarity

The results support the rationale for multimodal gait recognition based on modality-specific learning. IMU signals mainly encode segment-level motion trajectories, whereas EMG signals provide information about neuromuscular activation. This physiological complementarity helps explain why DSAF outperformed both single-modality variants and direct early fusion. The advantage of EMG is especially meaningful when similar kinematic trajectories conceal differences in motor-activation timing.

The ablation study also indicates that IMU remains the dominant modality for the current HuGaDB task. The strong IMU-only performance suggests that inertial sensing captures most of the information required for steady locomotion recognition. EMG-only performance was clearly weaker, which can be attributed to the sensitivity of surface EMG to electrode placement, skin-electrode impedance, local muscle recruitment variability, and amplitude scaling across subjects.

Nevertheless, EMG remains valuable when integrated with IMU through modality-specific encoding and adaptive fusion. Directly concatenating heterogeneous signals may cause the higher-dimensional IMU channels to dominate the representation, whereas the dual-stream design allows each modality to be encoded before fusion. The attention module further improves the representation by learning sample-dependent modality weights rather than using a fixed fusion rule. In this sense, EMG should be interpreted as a complementary cue rather than as a standalone dominant modality.

### 5.2 Recognition difficulty across activity categories

The class-wise results show that running was the easiest activity to recognize, whereas upstairs and downstairs were the most difficult classes. This pattern is reasonable because running produces distinctive temporal and amplitude characteristics in lower-limb inertial signals. In contrast, stair ascent and descent contain cyclic lower-limb patterns that partially resemble level walking, especially when analyzed in short fixed windows rather than complete task segments.

The distinction between ascending and descending stairs often depends on subtle differences in joint loading, vertical excursion, and muscle recruitment timing rather than large differences in gross limb trajectory. Subject-independent evaluation makes these distinctions harder because cadence, limb dominance, step height, and movement strategy can vary across participants. For similar reasons, posture transitions such as sit down and stand up remain more demanding than steady-state locomotion, although they were recognized more reliably than the two stair-related classes in the present results.

### 5.3 Subject-wise stability, reproducibility, and limitations

The added subject-wise LOSO analysis strengthens the interpretation of the aggregate results. In wearable recognition, a high mean score can be misleading if it is caused by one participant with unusually clean sensor placement, highly regular gait rhythm, or an easier class distribution. The 18-subject fold visualization shows that DSAF maintains a stable advantage across held-out participants, which supports the conclusion that the reported improvement reflects a more general cross-subject effect.

From a rehabilitation perspective, a model that remains robust under subject-independent evaluation is more informative for deployment than a model tuned to repeated measurements from the same user, because real applications often involve unseen patients or users. From an assistive-control perspective, the limited per-window inference time suggests that adaptive multimodal fusion may be feasible for online recognition pipelines in lower-limb exoskeletons, prosthetic interfaces, or wearable monitoring systems, provided that sensor synchronization and signal quality are maintained (Belal et al., 2024; Prasanth et al., 2021). Real-time gait phase estimation with wearable IMU data has also shown the importance of low-latency sequence modeling in online systems (Tang et al., 2024).

The revised preprocessing description also strengthens reproducibility. The manuscript now specifies the stored sampling rate, channel dimensions, channel meanings, window duration, LOSO partition rule, 15/2/1 training-validation-test subject split, fold-wise normalization rule, training protocol, and metric definitions. These details are essential because subject leakage, test-subject-specific normalization, or window-level random splitting can substantially inflate wearable-recognition performance.

External dataset validation. To examine whether the temporal inertial backbone generalized beyond HuGaDB, the IMU branch of DSAF was additionally evaluated on two independent inertial-sensor benchmarks. Because PAMAP2 and UCI-HAR do not provide synchronized quadriceps EMG channels comparable to HuGaDB, these experiments validate the IMU temporal encoder rather than the complete IMU-EMG fusion mechanism. On the PAMAP2 six-class matched task (walking, running, standing, sitting, upstairs, downstairs) under subject-wise LOSO evaluation, the DSAF-IMU branch achieved 94.36% accuracy and 93.78% macro-F1. On UCI-HAR (six official classes under subject-wise split), it reached 95.64% accuracy and 95.17% macro-F1. These results suggest that the temporal inertial backbone is not restricted to HuGaDB, whereas the specific benefit of EMG fusion should still be interpreted from the HuGaDB multimodal experiments.

This study also has several limitations. First, the complete IMU-EMG fusion model was evaluated on a single publicly available multimodal dataset (HuGaDB) with 18 healthy adult participants; although the IMU temporal backbone was externally checked on PAMAP2 and UCI-HAR, full multimodal validation with synchronized EMG remains necessary for clinical populations with neurological or musculoskeletal conditions. Second, the EMG configuration in HuGaDB covers only the quadriceps, which limits the representativeness of the neuromuscular signal (see Section 5.4 for a detailed discussion). Third, the attention weights analyzed in Section 4.3 may not fully reflect behavior on out-of-distribution clinical subjects. Fourth, the current model was evaluated offline; real-time deployment on embedded wearable hardware requires further optimization.

### 5.4 Limitations of single-muscle EMG in HuGaDB

The HuGaDB dataset includes only two surface EMG channels placed over the bilateral quadriceps (rectus femoris). While this configuration provides useful information about knee extensor activation during locomotion, it does not capture the full neuromuscular state of the lower limb. The quadriceps region alone cannot represent the activation of other major muscle groups involved in gait, including the hamstrings, gastrocnemius, tibialis anterior, and hip abductors. Furthermore, one EMG channel per quadriceps region cannot reliably characterize the composite activation of the entire quadriceps muscle group due to spatial selectivity limitations of surface electrodes. These constraints mean that the EMG contribution observed in the ablation study should be interpreted as a lower-bound estimate of neuromuscular benefit. Future work should evaluate DSAF with multi-site lower-limb EMG configurations to fully exploit neuromuscular information for clinical gait assessment and neuromotor rehabilitation applications.

The aggregate LOSO performance, subject-wise fold curves, class-wise metrics, confusion matrix, attention-weight visualization, and ablation results jointly suggest that the improvement is associated with both multimodal representation and adaptive fusion. The new error-distribution and attention analyses also clarify why stair negotiation and posture transitions remain more difficult than steady locomotion: these activities exhibit overlapping kinematic trajectories while relying more strongly on neuromuscular timing cues.

## 6 Conclusion

This study presented DSAF, a subject-independent multimodal gait-recognition framework that processes synchronized IMU and EMG signals through asymmetric modality-specific temporal encoders and combines them using physiological complementarity weighting. Under LOSO evaluation on HuGaDB, DSAF achieved 96.41% accuracy and 95.81% macro-F1, outperforming recent baselines with statistical significance. The ablation study confirmed that both modalities contribute complementary information, and that the attention mechanism provides consistent gains over naive fusion strategies. The subject-wise LOSO analysis demonstrated robust generalization across all 18 participants, whilst the external PAMAP2 and UCI-HAR checks suggested that the IMU temporal backbone also transfers to independent inertial-sensor benchmarks. These findings support the clinical potential of multimodal wearable sensing for subject-independent gait monitoring in rehabilitation and neuromotor assessment, whilst acknowledging that the single-muscle EMG configuration in HuGaDB limits the representativeness of neuromuscular information.

Compared with recent wearable-recognition baselines, DSAF achieved the best overall performance, reaching 96.41% accuracy, 96.08% macro-precision, 95.62% macro-recall, and 95.81% macro-F1. The added subject-wise LOSO figure showed that the improvement was not caused by one exceptionally easy subject, but was distributed across the 18 held-out participants. Class-wise analysis showed stable recognition across all eight activities, whereas upstairs and downstairs remained the most challenging categories. The ablation study further showed that IMU provides the main kinematic representation, EMG supplies complementary neuromuscular cues, and the combination of modality-specific dual-stream encoding with adaptive attention fusion yields the strongest overall result.

Overall, the study indicates that IMU and EMG can provide complementary information for subject-independent gait recognition. The proposed framework has potential value for wearable rehabilitation monitoring and assistive-control systems in which movement-state estimation and neuromuscular-intent information need to be jointly considered. Future work should validate the framework on larger and more diverse datasets and examine lightweight real-time deployment on embedded platforms.

## Data Availability

The original contributions presented in the study are included in the article/supplementary material, further inquiries can be directed to the corresponding author.
